# Editorial

**Published:** 1874-12

**Authors:** 


### Editorial.

### *"Preparatory Medicines."*

What are they? The *Sanitary Journal*, of Toronto, Canada, for September, quotes us at length, as condemning the use of "preparatory medicines," in language utterly regardless of orthography and syntax. The journal referred to is highly creditable to its editor, but needs a new proof reader. H.

### *"Not so Black as Painted."*

Under this caption the *Clinic* of November 14th, 1874, quotes from the CHICAGO MEDICAL JOURNAL of November, 1874, a short notice of Dr. Minor's new work on Child-bed Fever, which can scarcely be called complimentary. Unwilling to depend too much on a very retentive memory, we have searched the number of the CHICAGO MEDICAL JOURNAL indicated, and can find no notice whatever of the book in question. Now, we have sins enough of our own to answer for, without being made responsible for those of our neighbors, especially when they consist in criticisms upon "taste," themselves etymological blunders. Whatever we may have to say of the book in question, we shall not call it a "brochuoe," as we have still an old copy of "Spier and Surenne," a relic of school-boy days, which will protect us from such an exhibition of bad taste and ignorance. H.

The movement recently inaugurated in London to establish a memorial to the late Dr. Francis E. Anstie, has assumed, under the judicious management of those having it in charge, a most sensible direction. It is now designed to raise a fund which shall be applied to the education of his son, in the manner desired by his father, whose wishes in this regard have been so suddenly and unexpectedly frustrated by his untimely death. Dr. Anstie left his family, it appears, in somewhat straightened circumstances, and it is therefore fortunate that the custodians of this fund are endowed with a share of practical good sense, so rare in the present day of unfinished monuments to the forgotten dead. H.

---

***A Compilation of Returns of Vital Statistics** of the Chief Cities of the World, for the week mostly included within the first two weeks of September*, 1874.

| City. | Week ending | Deaths. | Rate per 1000 of Population. |
|---|---|---|---|
| Paris | September 18, 1874. | 720 | 20 |
| Brussels | " 12, 1874. | 91 | 29 |
| The Hague | " 12, 1874. | 33 | 18 |
| Amsterdam | " 12, 1874. | 106 | 19 |
| Copenhagen | " 10, 1874. | 120 | 31 |
| Christiana | " 15, 1874. | 25 | 18 |
| Berlin | " 12, 1874. | 467 | 29 |
| Breslau | " 5, 1874. | 147 | 34 |
| Vienna | " 12, 1874. | 257 | 21 |
| Rome | " 6, 1874. | 130 | 27 |
| Turin | " 6, 1874. | 85 | 21 (Annual.) |
| New York | August 29, 1874. | 618 | 31 " |
| Brooklyn | " 29, 1874. | 225 | 26 |
| Philadelphia | " 29, 1874. | 267 | 19 |
| Calcutta | " 1, 1874. | 204 | 24 (Annual.) |
| Bombay | " 25, 1874. | 265 | 21 |
| Madras | July 31, 1874. | 298 | 39 |
| London | September 19, 1874. | 1,242 | 19 (Annual.) |
| The United Kingdom (twenty-one cities) | " 19, 1874. | 3,383 | 23 (Annual.) |
| Edinburgh | " 19, 1874. | ............ | 21 |
| Glasgow | " 19, 1874. | ............ | 26 |
| Dublin | " 19, 1874. | ............ | 30 |
| Liverpool | " 19, 1874. | ............ | 36 |
| Chicago | " 12, 1874. | 230 | *29.9+ (Ann'l)<br>†26.58 " |
| " | " 19, 1874. | 188 | *24.44+ "<br>†21.72 " |

* Estimating the population at 400,000. † Estimating the population at 450,000.

There were about 200 births in Chicago, in each of the above weeks, as stated to us by Mr. J. W. Russell, the Secretary of the Board of Health.

The number of births in London for the week ending September 19, was 2,314. For the same period, in London and twenty other large towns of the United Kingdom, 5,407.

One of the deaths registered during the week ending September 19, 1874, in London, was of a servant whose age was stated to be 103 years ; the cause of death was certified as bronchitis. The death of a gentleman, on Sept. 12, was referred to "heart disease, accelerated by taking chloral hydrate." A little girl, aged seven, died, in Guy's Hospital, from hydrophobia.

The 1,242 deaths occurring in London, included 105 from scarlet fever, and not one from small-pox. Scarlet fever is increasing, and prevails fatally in Kensington Town, in Dublin, and in eighteen large English towns.

